# A UK prospective multicentre decision impact, decision conflict and economic evaluation of the 21-gene assay in women with node+ve, hormone receptor+ve, HER2-ve breast cancer

**DOI:** 10.1038/s41416-024-02588-9

**Published:** 2024-02-02

**Authors:** Simon Holt, Mark Verrill, Laura Pettit, Anna Rigg, Tamas Hickish, Caroline Archer, Jo Dent, Marianne Dillon, Mark Nathan, Ludger Barthelmes, Shazza Rehman, Yousef Sharaiha, Paige Innis, Priya Sai-Giridhar, Saira Khawaja

**Affiliations:** 1 Swansea University, Swansea, UK; 2 Northern Centre for Cancer Care, Newcastle, UK; 3 Royal Shrewsbury Hospital, Shrewsbury, UK; 4 Guy’s and St Thomas’ Hospitals, London, UK; 5 University Hospitals Dorset, Bournemouth, UK; 6 Portsmouth Hospitals University NHS Trust, Portsmouth, UK; 7 Huddersfield NHS Trust, Huddersfield, UK; 8 York Hospital, York, UK; 9 Cumberland Infirmary, Carlisle, UK; 10 Airedale NHS Foundation Trust, Keighley, UK; 11 Prince Philip Hospital, Llanelli, UK; 12 Exact Sciences Corporation, Redwood City, CA USA

**Keywords:** Breast cancer, Molecular medicine, Predictive markers, Cancer genomics, Gene expression

## Abstract

**Background:**

For a tumour profiling test to be of value, it needs to demonstrate that it is changing clinical decisions, improving clinical confidence, and of economic benefit. This trial evaluated the use of the Oncotype DX Breast Recurrence Score® assay against these criteria in 680 women with hormone receptor-positive (HR+), HER2-negative early breast cancer with 1–3 lymph nodes positive (LN+) in the UK National Health Service (NHS).

**Methods:**

Prior to receipt of the Recurrence Score (RS) result, both the physician and the patient were asked to state their preference for or against chemotherapy and their level of confidence on a scale of 1–5. Following receipt of the RS result, the physician and patient were asked to make a final decision regarding chemotherapy and record their post-test level of confidence.

**Results:**

Receipt of the RS result led to a 51.5% (95% CI, 47.2–55.8%) reduction in chemotherapy, significantly increased the relative and absolute confidence for both physicians and patients and led to an estimated saving to the NHS of £787 per patient.

**Conclusion:**

The use of the Oncotype DX assay fulfils the criteria of changing clinical decisions, improving confidence and saving money.

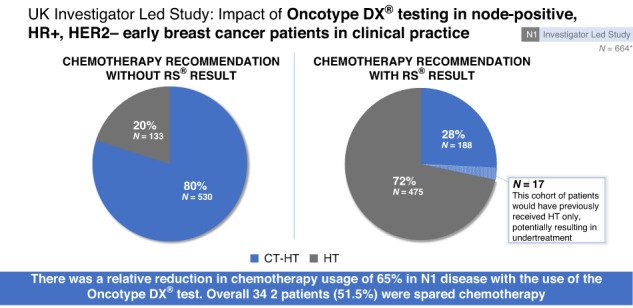

## Introduction

The Oxford overview of breast cancer published in 2005 [1] demonstrated a 14.6% benefit at 5 years in terms of recurrence from adjuvant chemotherapy (CT) in pre-menopausal women and a 5.9% benefit in postmenopausal women. Consequently, most patients with early node positive breast cancer treated with adjuvant chemotherapy will not benefit (94.1% of post-menopausal and 85.4% of pre-menopausal).

Clearly, the unnecessary use of chemotherapy in women with early breast cancer constitutes a significant physical and economic burden to the patients and a significant economic burden to the NHS [2].

The Oncotype DX Breast Recurrence Score® assay (Exact Sciences Corporation, Madison, WI, USA) is a 21-gene breast cancer assay for women with hormone receptor-positive (HR+), HER2-negative (HER2−) breast cancer which returns a Recurrence Score (RS) result of between 0 and 100. As the RS result increases, the risk of recurrence also increases. Several prospective-retrospective trials have demonstrated the value of the assay in node-negative breast cancer and most recently the prospective TAILORx Trial [3] demonstrated that postmenopausal women with a score of 25 or lower did not benefit from the addition of chemotherapy to hormone therapy in terms of invasive disease-free survival, distant recurrence, or overall survival. In pre-menopausal women there was no benefit with a score of 15 or lower, although there was a small benefit with a score of 16–25.

However, about one in three women presenting with HR+, HER2− breast cancer have lymph node involvement [4]. At the time of initiation of our trial, there was evidence to suggest that the RS result could help identify some women with early breast cancer and 1–3 nodes involved who will not benefit from the addition of chemotherapy (TransATAC [5], SWOG-8814 [6], PACS-01 [7], NSABP B-28 [8], WSG PlanB [9, 10] and registry data from CLALIT [11] and SEER [12]). In TransATAC, the results showed the confidence intervals of the curves for node-negative and 1–3 node-positive patients when plotting RS result versus the 9-year risk of distant recurrence, overlapped below a score of 18. A prospective/retrospective analysis of the SWOG-8814 chemotherapy trial of postmenopausal women showed that patients with a recurrence score of less than 18 had no benefit from the addition of chemotherapy, but that there was a statistically significant benefit if the score was 31 or higher. During our trial, the RxPONDER trial [13] reported some preliminary results which showed that postmenopausal women with an RS result of 25 or lower showed no benefit in terms of invasive disease-free survival, new primary breast cancer or death from the addition of chemotherapy. Conversely, in pre-menopausal women, chemotherapy conferred an invasive disease-free survival and distant relapse-free benefit at 5 years independent of the RS result.

NCCN invasive breast cancer guidelines [14] published in 2023 say, “The 21-gene assay (Oncotype DX®) is preferred by the NCCN Breast Cancer Panel for prognosis and prediction of chemotherapy.” For postmenopausal node positive (1–3 nodes), the NCCN category of evidence and consensus is level 1. For pre-menopausal patients, the level of evidence is considered to be 2A. The ASCO breast cancer guidelines [15] recommend that clinicians may use Oncotype DX, MammaPrint, Breast Cancer Index (BCI), and EndoPredict to guide adjuvant endocrine and chemotherapy in patients who are postmenopausal (or aged 50 years and older if the menopausal status is unknown) with early-stage ER+ , HER2– breast cancer that is node-negative or with 1–3 positive nodes.

It was unknown whether the use of Oncotype DX test in women with HR+, HER2− breast cancer and 1–3 nodes positive treated by the UK National Health Service (NHS) would lead to a reduction in use of chemotherapy and whether it would increase or decrease confidence in the use or avoidance of chemotherapy for oncologists and patients. Similarly, it was unclear whether the use of Oncotype DX test would prove cost-effective. However, similar studies performed prior to the reporting of RxPONDER conducted in Canada (which has a publicly funded healthcare system with similar approaches in health technology-based decisions) showed a change in treatment decisions of 36% away from chemotherapy [16]. The trial also demonstrated improved physician and patient confidence in the post-Oncotype DX recommendations. A second study published after RxPONDER reported a 27–67% reduction in chemotherapy recommendations in Canada and a review of the world literature showed a reduction of between 18 and 69% [17].

This trial was conducted in a cross-section of UK NHS hospitals and was designed to measure the decision impact of using Oncotype DX test in women with HR+, HER2− breast cancer and 1–3 nodes positive and to measure whether its use increased or decreased the confidence of oncologists and their patients in the final decision on whether chemotherapy should be used. An economic assessment of the use of Oncotype DX test in these patients was also carried out.

## Methods

The trial method and documentation, including the patient information leaflets and consent process, were approved by the Wales REC 7 Research Ethics Committee, as was the substantial amendment issued shortly after the RxPONDER trial reported its initial findings part way through the recruitment period (December 2020). The trial was funded by the Prince Philip Hospital Breast Care Unit Charity, and the Oncotype DX tests were provided by Exact Sciences Corporation.

Having completed surgical treatment, women over the age of 18 with HR+ and HER2− breast cancer and either micrometastases or 1–3 lymph nodes involved were eligible to join the trial. The patients needed to have an adequate performance status (ECOG 0 or 1) and needed to be fit and willing to undergo chemotherapy, if indicated. Patients with a prior history of breast cancer in the same breast, multicentric or microinvasive disease, evidence of metastatic disease or who had received prior neo-adjuvant chemotherapy were not eligible. Patients with current medical or psychiatric conditions which would impair their ability to provide personal informed consent were excluded. Patients who had received neo-adjuvant endocrine treatment were included, provided the Oncotype DX test was performed on the diagnostic core biopsy obtained prior to treatment initiation.

Patients were recruited from 14 centres (3 teaching hospitals, 2 regional cancer centres and 9 district general hospitals). Potential candidates were identified at the post-surgery multi-disciplinary team (MDT) meeting and invited to join the trial once the final histology report was available. Consent was obtained according to the principles of Good Clinical Practice and the patient then registered with the trial centre.

Adequate nodal assessment was defined as a minimum of three nodes where one node was involved (to conform with the Giuliano criteria), or an axillary dissection in patients with two or three nodes involved. Since this often meant a patient having to undergo a second axillary procedure which became problematic during the COVID-19 epidemic, this requirement was relaxed during 2020.

Details on menopausal status were recorded where known; otherwise, as in the RxPONDER trial analysis, women aged 49 and under were recorded as pre-menopausal and women 50 or over as post-menopausal.

On entry to the trial, the oncologist and patient were asked to specify their treatment preference (CT+ Hormone Therapy [HT] or HT only) and their degree of confidence in their treatment decision using a five-point scale (1–5) from strongly disagree to strongly agree. Patients were able to choose “Uncertain” (0), but the oncologist had to express a view using the five-point scale. The oncologist was asked to specify the type of chemotherapy they would give and the hormone treatment they would select, and to state if ovarian suppression was to be used in addition.

The Oncotype DX test was then ordered, and once the RS result was reported the oncologist and patient reviewed the result together and recorded a final decision on treatment. Again, they were asked to rate their degree of confidence on their final decision using the same 5-point scale.

The RxPONDER trial reported its early findings part way through this trial. Since this may have influenced chemotherapy recommendations, post hoc analyses of the pre- and post-RxPONDER cohorts were performed separately (overall and for post-menopausal women) using recruitment at 1 Jan 2021 as the cut off. A substantial amendment to the trial was issued in early 2021 bringing investigators attention to the RxPONDER findings, but following a full discussion with the ethics committee, it was decided not to close this trial to pre-menopausal women as the benefits of chemotherapy remain small (a 5-year IDFS difference of 4.9%) and there may be special circumstance where treatment decisions could still be influenced by the RS result. It is also uncertain how much of the chemotherapy benefit demonstrated by RxPONDER might relate to the induction of an early menopause which could be simulated by using ovarian suppression as an alternative.

The economic analysis is based on the estimates of UK chemotherapy costs in early breast cancer contained in the comprehensive supplementary tables of Berdanov et al. [18]. These costs are an aggregate of drug costs listed in the British National Formulary or the Drugs and Pharmaceutical Electronic Marketing Tool (eMIT), administration costs taken from NHS reference costs SB15Z, the cost of treating complications of chemotherapy derived from HRGs (where stated) and corrected for the percentage of different drug regimens used in early breast cancer informed by expert opinion.

### Statistical analysis

Descriptive statistics were used to summarise patient and tumour characteristics. Wilcoxon rank-sum tests were used to compare continuous variables by RxPONDER cohorts and Chi-square tests or Fisher’s Exact tests were used to compare categorical variables by RxPONDER cohorts.

The relative change in chemotherapy was calculated as the difference in the proportion of patients with chemotherapy recommendations post-assay vs. pre-assay. The absolute change in chemotherapy was calculated as the difference in the proportion of patients with chemotherapy recommendations post-assay vs. pre-assay divided by the pre-assay proportion. McNemar’s test was used to compare the proportion of patients recommended chemo-hormonal therapy pre- vs. post-assay (overall, by RS result, by RxPONDER cohort, and for post-menopausal patients). The proportion (and 95% CIs) of patients who received a change in treatment pre- vs. post-assay overall, by RS result, and the number of nodes involved were estimated using Exact tests. The Mantel–Haenszel Chi-square test was used to evaluate post-assay treatment received in the pre- vs. post-RxPONDER cohorts.

The absolute change in confidence from pre- to post-assay was calculated by subtracting the post-assay confidence from the pre-assay confidence. A positive result indicated increased confidence, whereas a negative result indicated decreased confidence. The Wilcoxon signed-rank test was used to compare confidence pre- vs. post-assay. The relative change in confidence as derived by multiplying the number of patients in each change category by the number of increased/decreased levels in confidence. The average change in confidence post-assay was calculated by summing the relative confidence. The Cochran–Mantel–Haenszel test was used to compare the average change in confidence from pre- vs. post-assay between patients recruited before vs. after RxPONDER results release. *P* values < 0.05 were considered statistically significant.

Among node-positive decision impact studies, the change rate of treatment recommendations ranged from 24 to 51% (Oratz R et al. [19], Torres et al. [16]). The sample size justification for this study is based upon anticipating an overall treatment change rate of 26%, which is a conservative estimate given the unknown expected change amongst individual nodal groups. Based on this assumed change rate, a sample size of 60 patients is required for a 95% CI of 15.5–38.6% (a CI width of 23.1%). The larger sample size of this study allowed for decision-impact subgroup analyses.

## Results

### Recruitment patterns

Fourteen centres were involved in the trial, each recruiting between 4 and 84 participants (see Appendix Fig. 1 for details). Recruitment began on October 30, 2017 and closed on March 31, 2022, a 4-year and 5-month period. Of the 680 patients recruited, 16 (2.4%) patients were excluded from the final analysis: 3 patients were confirmed HER2 positive on subsequent testing, 3 patients could not attend their follow-up assessment or moved away, 5 specimens were insufficient for testing, 2 patients withdrew consent, and 2 patients were found to have more extensive disease (1 bone metastases; 1 a second breast primary).

The final analysis population consisted of 664 patients (450 enrolled prior to RxPONDER reporting in December 2020 vs. 214 after).

The accrual rate of patients was relatively steady at about 40 patients per quarter with a dip to 25 patients during the quarter of the first COVID-19 lockdown, suggesting that clinicians were recruiting most of the eligible patients from the outset of the trial (Appendix Fig. 2).

### Demographics and case mix

Patient and tumour characteristics, including the distribution of the RS results, are summarised in Table 1, overall and by whether a patient completed the study during the pre- vs. post-RxPONDER period.

**Table 1:** Patient and tumour characteristics for overall, pre- and post-RxPONDER cohorts.

Characteristics		Overall (*n* = 664)	Pre-RxPONDER Cohort^a^ (*n* = 450)	Post-RxPONDER Cohort^a^ (*n* = 214)	*P* value^b^
Age (years)	*N*	664	450	214	
	Mean (SD)	58.2 (10.6)	57.5 (11)	59.7 (9.3)	0.011
	Median (Q1, Q3)	58 (51, 66)	57 (49, 66)	59 (53, 67)	
	Min, Max	27, 85	27, 81	34, 85	
Age (years), *N* (%)	<40	23 (3.5%)	19 (4.2%)	4 (1.9%)	0.008
	40-49	117 (17.6%)	94 (20.9%)	23 (10.7%)	
	50-59	232 (34.9%)	147 (32.7%)	85 (39.7%)	
	60-69	176 (26.5%)	115 (25.6%)	61 (28.5%)	
	70+	116 (17.5%)	75 (16.7%)	41 (19.2%)	
Menopausal status, *N* (%)	Pre	152 (22.9%)	114 (25.3%)	38 (17.8%)	0.030
	Post	512 (77.1%)	336 (74.7%)	176 (82.2%)	
Histological subtype, *N* (%)	Ductal	526 (79.2%)	370 (82.2%)	156 (72.9%)	0.006
	Lobular	89 (13.4%)	58 (12.9%)	31 (14.5%)	
	Mixed	23 (3.5%)	12 (2.7%)	11 (5.1%)	
	Papillary	10 (1.5%)	4 (0.9%)	6 (2.8%)	
	Mucinous	7 (1.1%)	2 (0.4%)	5 (2.3%)	
	Tubular	4 (0.6%)	3 (0.7%)	1 (0.5%)	
	Pleomorphic Lobular	3 (0.5%)	1 (0.2%)	2 (0.9%)	
	Medullary	2 (0.3%)	0 (0.0%)	2 (0.9%)	
Tumour size (mm)	*N*	664	450	214	
	Mean (SD)	29.12 (20.95)	29.35 (22.47)	28.64 (17.39)	0.40
	Median (Q1, Q3)	24 (18, 31.5)	24 (17, 31)	25 (18, 33)	
	Min, max	2, 180	2, 180	5, 150	
Tumour size (mm), *N* (%)	0 to <20	214 (32.2%)	151 (33.6%)	63 (29.4%)	0.57
	20 to <40	333 (50.2%)	219 (48.7%)	114 (53.3%)	
	40 to <60	72 (10.8%)	46 (10.2%)	26 (12.1%)	
	60 to <80	26 (3.9%)	18 (4.0%)	8 (3.7%)	
	80 to <100	6 (0.9%)	5 (1.1%)	1 (0.5%)	
	100+	13 (2.0%)	11 (2.4%)	2 (0.9%)	
Grade, *N* (%)	G1	82 (12.3%)	55 (12.2%)	27 (12.6%)	0.084
	G2	412 (62.0%)	291 (64.7%)	121 (56.5%)	
	G3	170 (25.6%)	104 (23.1%)	66 (30.8%)	
Nodes involved, *N* (%)	Micrometastases	26 (3.9%)	26 (5.8%)	0 (0.0%)	0.002
	1	384 (57.8%)	249 (55.3%)	135 (63.1%)	
	2	191 (28.8%)	128 (28.4%)	63 (29.4%)	
	3	63 (9.5%)	47 (10.4%)	16 (7.5%)	
ECOG status^c^, *N* (%)	0	541 (81.5%)	369 (82.0%)	172 (80.4%)	0.28
	1	70 (10.5%)	50 (11.1%)	20 (9.3%)	
	Missing	53 (8.0%)	31 (6.9%)	22 (10.3%)	
Recurrence score	*N*	664	450	214	
	Mean (SD)	17.5 (10.8)	17.2 (10)	18.2 (12.4)	0.72
	Median (Q1, Q3)	16 (11, 22)	16 (11, 21)	16 (10, 22)	
	Min, Max	0, 78	0, 75	0, 78	
RS category, *N* (%)	0–13	261 (39.3%)	176 (39.1%)	85 (39.7%)	0.51
	14–25	305 (45.9%)	212 (47.1%)	93 (43.5%)	
	26–100	98 (14.8%)	62 (13.8%)	36 (16.8%)	
RS category, *N* (%)	0–25	566 (85.2%)	388 (86.2%)	178 (83.2%)	0.30
	26–100	98 (14.8%)	62 (13.8%)	36 (16.8%)	

^a^Pre-RxPONDER and Post-RxPONDER refer to patients recruited prior to and after the release of the RxPONDER results, respectively.

^b^*P* values were derived from Wilcoxon rank-sum tests for continuous variables and Chi-square tests or Fisher’s Exact test for categorical variables.

^c^ECOG status 0 = Fully active, no performance restrictions; 1 = Strenuous physical activity restricted; fully ambulatory and able to carry out light work.

The median age at registration was 58 years (range, 27–85), and 77.1% were post-menopausal. Women in the post-RxPONDER cohort were more likely to be post-menopausal (82.2%, *P* = 0.03). A detailed age distribution, overall and by RxPONDER cohort, are available in Appendix Fig. 3. The majority of patients had a RS result of 0–25 (*n* = 566, 85.2%); while 98 (14.8%) had a RS result of 26–100. RS result did not differ significantly between RxPONDER cohorts (*P* = 0.72, Table 1). Details of RS results are available in Appendix Table 1. Distribution of the final chemotherapy decision by RS result are available in Appendix Figs. 4–6.

Initially, micrometastases were included in the trial but following the decision of NICE to include these patients for routine testing as with node-negative patients, this arm was closed in September 2019 after 26 patients had been recruited. Overall, 384 (57.8%) patients with one positive node, 191 (28.8%) with 2 positive nodes and 63 (9.5%) with 3 positive nodes were included (Table 1). There was a significant difference in the number of nodes involved between the RxPONDER cohorts. In the pre-RxPONDER cohort, 26 (5.8%) had micrometastases, 249 (55.3%) had 1 node, 128 (28.4%) had 2 nodes, and 47 (10.4%) had 3 nodes involved, whereas 135 (63.1%) of patients in the post-RxPONDER cohort had one node, 63 (29.34%) had 2 nodes, and 16 (7.5%) had 3 nodes involved (*P* = 0.002, Table 1).

The ECOG Status of patients is summarised in Table 1 and demonstrates that the majority of patients (*n* = 541, 81.5%) had an ECOG status of 0 and 70 (10.5%) had an ECOG status of 1.

### Decision impact results

The final chemotherapy decisions by oncologists are summarised in Table 2. Overall, there was a 51.5% (95% CI: 47.2–55.8) absolute reduction and 64.5% (95% CI: 60.2–69.1) relative reduction in chemotherapy following the reporting of the RS result. Oncologists chose to stay with their initial decision to give CT + HT in 171 cases (25.8%) and to stay with their original decision to give HT-only in 117 cases (17.6%). Oncologists changed their decision from CT + HT to HT therapy alone in 359 cases (54.1%) and changed from HT-only to CT + HT in 17 cases (2.6%) (*P* < 0.001).

**Table 2:** Oncologists’ decision impact results overall, by RxPONDER, by RS result, and by post-menopausal RxPONDER cohorts.

	Decision impact, *N* (%)	CT + HT unchanged	HT-only unchanged	CT + HT to HT-only	HT-only to CT + HT	Total *N*	*P* value^a^
	Overall	171 (25.8)	117 (17.6)	359 (54.1)	17 (2.6)	664	<0.001
RxPONDER cohort	Pre	122 (27.1)	78 (17.3)	241 (53.6)	9 (2.0)	450	<0.001
	Post	49 (22.9)	39 (18.2)	118 (55.1)	8 (3.7)	214	<0.001
Recurrence score	0–13	13 (5.0)	68 (26.1)	178 (68.2)	2 (0.8)	261	<0.001
	14–25	72 (23.6)	48 (15.7)	178 (58.4)	7 (2.3)	305	<0.001
	26–100	86 (87.8)	1 (1.0)	3 (3.1)	8 (8.2)	98	0.13
Post-menopausal, Pre-RxPONDER cohort	0–25	40 (13.7)	57 (19.5)	194 (66.4)	1 (0.3)	292	<0.001
	26–100	36 (81.8)	1 (2.3)	2 (4.6)	5 (11.4)	44	0.26
Post-menopausal, Post-RxPONDER cohort	0–25	2 (1.4)	36 (24.5)	105 (71.4)	4 (2.7)	147	<0.001
	26–100	28 (96.6)	0 (0.0)	0 (0.0)	1 (3.5)	29	n/a

*CT* chemotherapy, *HT* hormone therapy.

^a^*P* value derived from McNemar’s test evaluating the patients who changed treatment recommendation pre- vs. post-assay.

In the pre-RxPONDER cohort, there was a significant proportion of patients whose treatment recommendation changed from CT + HT to HT-only post-assay, *N* = 241 (53.6%) (*P* < 0.001). Similarly, in the post-RxPONDER cohort, there was a significant proportion of patients whose treatment recommendation changed from CT + HT to HT-only post-assay, *N* = 118 (55.1%) (*P* < 0.001). The equivalent tables for the patients’ decision impact are contained in Appendix Table 2.

In the 0–13 RS group, oncologists changed their decision from CT + HT to HT-only in 178 (68.2%) patients. In the 14–25 RS group, oncologists chose to stay with their initial decision to give CT + HT in 72 (23.6%) patients and changed their decision from CT + HT to HT-only in 178 (58.4%). Meanwhile, in the 26–100 RS group, oncologists chose to stay with their initial decision of CT + HT in 86 (87.8%) of patients. The proportion of patients whose treatment recommendation changed from CT + HT to HT-only post-assay by RS result is presented in Table 2. This reduction in chemotherapy is consistent when analysed by RS or the number of nodes involved (Appendix Fig. 7-8).

In pre-RxPONDER, post-menopausal women with a RS result of 0–25, 194 (66.4%), oncologists changed their treatment recommendation from CT + HT to HT-only (*P* < 0.001). In post-RxPONDER, post-menopausal women with a RS result of 0–25, 105 (71.4%), oncologists changed their treatment recommendation from CT + HT to HT-only (*P* < 0.001). By contrast, oncologists maintained their recommendation for CT + HT post-assay in 36 (81.8%) and 28 (96.6%) of patients with an RS result of 26–100 in the post-menopausal, pre- and post-RxPONDER cohorts, respectively. Among post-menopausal patients with a RS result below 26, there was a significant reduction in the proportion of patients receiving CT + HT in the post-RxPONDER cohort vs. those in the pre-RxPONDER cohort (14.0% pre-RxPONDER vs. 4.1% post-RxPONDER, *P* = 0.002) (Table 3).

**Table 3:** Oncologist’s post-assay treatment recommended in post-menopausal women with RS result of 0–25 in pre- v post-RxPONDER cohorts.

	Post-assay treatment	
RxPONDER cohort	HT-only	CT + HT
Pre (*n* = 292)	251 (86.0%)	41 (14.0%)
Post (*n* = 147)	141 (95.9%)	6 (4.1%)

Treatment received post-assay for pre-RxPONDER vs post-RxPONDER, Mantel–Haenszel Chi-square test *P* = 0.002.

The details of the patient decision impact results are reported in Appendix Table 2 and mirror the findings reported for the oncologists.

### Decision conflict results

A summary of the absolute change in the confidence of both the oncologists and patients is shown in Table 4. In 55% of cases, the addition of the RS results added confidence to the oncologists’ decision regarding chemotherapy, even if the treatment recommendation was changed (*P* < 0.001). In 12% of cases, the oncologist became less confident, and in 33% of cases, the level of confidence did not change. In the case of the patients, the levels of confidence were improved even more, with 71% becoming more confident, even if the treatment decision was changed (*P* < 0.001). In 7%, they became less confident and in 22% their confidence was unchanged.

**Table 4:** Absolute change in confidence in treatment choice for oncologists and patients from pre- to post-assay.

	Oncologist (*N*, %)^a^	Patient (*N*, %)^b^
Increased confidence	365 (55.0)	465 (70.9)
Unchanged	221 (33.3)	147 (22.4)
Decreased confidence	78 (11.8)	44 (6.7)
Total	664 (100)	656* (100)

^a^Change in confidence pre- to post-assay for oncologists, Wilcoxon signed-rank test *P* = < 0.001.

^b^Change in confidence pre- to post-assay for Patients, Wilcoxon signed-rank test *P* = < 0.001.

*Eight patients were excluded owing to missing post-assay questionnaires.

The relative change in confidence for oncologists is shown in Table 5 overall and by RxPONDER cohort. Overall, confidence was improved by +0.75 out of possible four levels. There was an increased level of confidence following the reporting of RxPONDER from +0.70 to +0.86, but this was not statistically significant (*P* = 0.10, Table 5).

**Table 5:** Relative change in confidence in treatment choice for oncologists from pre- to post-assay (overall and by RxPONDER cohorts).

Oncologist	Overall		Pre-RxPONDER Cohort		Post-RxPONDER cohort	
Decision conflict	*N*	Shift	*N*	Shift	*N*	Shift
+4	17	68	11	44	6	24
+3	46	138	29	87	17	51
+2	90	180	60	120	30	60
+1	212	212	144	144	68	68
0	221	0	143	0	78	0
−1	59	−59	46	−46	13	−13
−2	16	−32	15	−30	1	−2
−3	3	−9	2	−6	1	−3
−4	0	0	0	0	0	0
Total	664	498	450	313	214	185
Average change		**+0.75**		**+0.70**		**+0.86**

Change in physician confidence pre- to post-assay for pre-RxPONDER vs post-RxPONDER, Cochran–Mantel–Haenszel Statistic *P* = 0.10.

Bold values emphasise the final conclusion of the table.

The relative change in confidence for patients is shown in Table 6 (overall and for pre- and post-RxPONDER cohorts). Overall, confidence was improved by +2.43 out of possible five levels. There was an increased level of confidence following the reporting of RxPONDER from +2.32 to +2.68 (*P* = 0.05, Table 6).

**Table 6:** Relative change in confidence for patients from pre- to post-assay (overall and by RxPONDER cohorts).

Patient change	Overall		Pre-RxPONDER cohort		Post-RxPONDER cohort	
Decision impact	*N*	Shift	*N*	Shift	*N*	Shift
+5	177	885	125	625	52	260
+4	136	544	71	284	65	260
+3	27	81	17	51	10	30
+2	24	48	18	36	6	12
+1	101	101	75	75	26	26
0	147	0	106	0	41	0
−1	31	−31	22	−22	9	−9
−2	10	−20	6	−12	4	−8
−3	1	−3	1	−3	0	0
−4	2	−8	2	−8	0	0
−5	0	0	0	0	0	0
Totals	656*	1597	443	1026	213	571
Average change		**+2.43**		**+2.32**		**+2.68**

Change in patient confidence pre- to post-assay for pre-RxPONDER vs post-RxPONDER, Cochran–Mantel–Haenszel Statistic *P* = 0.05.

*Eight patients were excluded owing to missing post-assay confidence score.

Bold values emphasise the conclusion of the table.

### Economic analysis results

Using the estimates of Berdunov et al. [18], the average cost of a course of chemotherapy in the UK is £6000–7000. An estimate of the overall cost saving of 344 courses is £2,064,000–2,408,000 and the overall cost of 664 Oncotype DX tests at the list price of about £2580 (although an undisclosed discount applies to the NHS) is £1,713,120. Thus, the use of Oncotype DX test represents a significant saving of about £787 per patient to the NHS.

In the postmenopausal post RxPONDER cohort of 176 patients, 101 patients were spared chemotherapy after testing which represents a more significant saving of about £1150 per patient.

The personal and societal savings are significantly higher when the costs of work absence (c. £4000 per working person), additional care costs borne by family and friends and out-of-pocket expenses for the patient such as travel costs, wigs and over-the-counter medications (c. £1100) are taken into account [2].

## Discussion

The population of women included in this trial represent a good cross-section of the general population of women presenting in the UK and derive from a mixture of rural, urban, and teaching hospital practices. Therefore, the results can be expected to reflect clinical practice throughout the UK.

Very little of the required data was missing, giving the trial good reliability.

Although the initial protocol included a cohort of patients with nodal micrometastases, NICE guidance was changed to allow reimbursed Oncotype DX testing of this group shortly after the start of the trial. Recruitment was closed early after 26 (of a proposed 70) cases were included. These cases have been retained in the final analysis.

The trial was confounded by two events which lead to some necessary changes in the protocol. The first of these was the COVD-19 pandemic which, in some cases, delayed admission to hospital and lead to more neo-adjuvant endocrine therapy being prescribed. This in turn changed the routine of sending the surgical specimen for Oncotype DX testing to sending the diagnostic core instead. In practice, this did not affect the RS result but did lead to five patients being excluded because there was insufficient tumour to test.

The COVID-19 pandemic also reduced the availability of operating time and increased the risk to the patient of admission to hospital. This changed the practice of offering further axillary surgery to patients with positive nodes following sentinel node biopsy. Initially, the protocol demanded that adequate assessment of the axilla be required for entry to the trial, but this had to be revised in the light of the changed clinical circumstances and 38 patients who would have been excluded at the start of the trial were subsequently included. This accords with the recruitment policy of the RxPONDER trial which had no set criteria for adequate axillary assessment.

The second confounding factor was the reporting early of the preliminary results of the RxPONDER trial which studied the use of Oncotype DX to inform chemotherapy benefit in node-positive breast cancer. This report was presented at the San Antonio Breast Cancer Symposium as an oral abstract in December 2020. The trial concluded that postmenopausal women with an RS result of 25 or less did not significantly benefit from CT in addition to HT. It also concluded that there was a benefit to chemotherapy in all pre-menopausal women whatever the RS result.

These findings were disseminated promptly to all recruitment centres and lead to a major protocol amendment. However, after careful consideration with all the stakeholders and reconsideration by the ethics committee, it was decided not to close the pre-menopausal recruitment arm. The reasoning behind this decision was based on remaining uncertainties about the details of the RxPONDER trial in the absence of a published paper (which subsequently appeared on Dec 1, 2021 [13]) and contradictory evidence available elsewhere [5–12] which suggested there may still be some benefit of Oncotype DX testing in pre-menopausal women particularly if ovarian suppression is offered as an alternative to chemotherapy. However, reporting of this trial would be expected to alter clinical practice and prompted a post hoc analysis of the data by pre- and post-RxPONDER cohorts, even though the trial was not initially designed to demonstrate such a difference.

As expected, the number of pre-menopausal patients registered after the RxPONDER trial reported decreased by 8.3% from 25.3 to 17.8%. (Table 1).

Review of the patient demographics show that they reflect the expected features seen in the general UK breast cancer population, suggesting that there was no significant selection bias in the trial population. The only feature of note is the relative underrepresentation of grade 1 tumours, (12.3% as opposed to an expected c.20% in the overall breast cancer population). The likely explanation for this is that grade 1 tumours are less likely to metastasise to the lymph nodes and accords with other analyses such as that of the SEER database [20].

The ECOG status shows the population were generally fit as would be expected given the entry criteria.

### Decision impact discussion

Analysis of the decision impact results shows that of the initial 530 patients selected for adjuvant chemotherapy, after the RS result became available, only 188 patients finally required treatment, representing a 65% relative reduction (95% CI: 60.2–69.1). Seventeen of the 134 (12.7%) patients initially selected for HT-only returned an unexpectedly high RS result, making chemotherapy necessary. Thus, 342 out of 664 patients were spared chemotherapy (51.5%, 95% CI: 47.2–55.8%). This highly significant reduction in chemotherapy post-assay is observed when broken down by RS result and number of nodes involved (Appendix Figs. 7 and 8). This is in line with the findings in other healthcare systems similar to the NHS (Hassan 2020, Canada [21]; Mattar 2021, Brazil [22] and Loncaster 2017, UK [23]), although there are several studies summarised in Yordanova [17] showed lower impacts of between 18% and 45% in other healthcare systems.

The decision impact results follow the expected pattern when broken down by the RS cut-offs used by RxPONDER (0–13, 14–25, 26–100) (Table 2).

However, looking at the post-menopausal population, there is a highly significant reduction in the use of chemotherapy in patients with an RS below 26 in the post-RxPONDER cohort (*P* = 0.002, Table 3). This suggests that the oncologists involved in this trial were quick to respond to the findings of the RxPONDER trial.

Within the trial population, there were 11 women (average age 60.5 years) who were initially recommended HT alone but who returned an unexpectedly high RS and had to be changed to CT + HT. Older women with high recurrence scores are known to do particularly badly without chemotherapy as demonstrated by the SEER data. Although small in number, these women are an important group to identify as without chemotherapy, they are at high risk of recurrence with the associated increased costs of further treatment.

### Decision conflict discussion

Decision conflict arises when the degree of certainty about the correct course of treatment is reduced by an intervention. However, the same intervention may also clarify the decision and make the clinician or patient more comfortable with the outcome even if it is changed.

Despite over half of patients having their recommended treatment changed, these results clearly demonstrate that the use of the Oncotype Dx improved the confidence of both the physicians and the patients (oncologists’ absolute increase in confidence 55%, relative increase in confidence +0.75, patients’ absolute increase in confidence 71%, relative increase +2.43; Tables 5 and 6). There was an initial concern that changing the treatment recommendation after the return of the RS result might undermine the confidence of the oncologists in their clinical judgement leading to an awkward situation with the patient and that it might decrease the patient’s confidence in her physician. However, these concerns have been dispelled.

In the case of the patients, the reporting of the RxPONDER trial improved their relative confidence from +2.32 to +2.68 which is statistically significant (*P* = 0.05, Table 6). However, statistical significance is not achieved in the case of the oncologist, where the increase in relative confidence was +0.69 to +0.86 (*P* = 0.10, Table 5), but this increase in confidence hints that RxPONDER has had a positive impact.

In this study, the oncologist’s confidence was increased after reporting of the Oncotype DX result in 55%, unchanged in 33% and decreased in 12% of cases. These findings are in line with those reported by Torres [16] (improved in 49%, unchanged in 40% and decreased in 10%).

The patients in this trial expressed increased confidence in 77%, unchanged in 22% and decreased in 7% of cases. The equivalent results report by Torres were increased in 54%, unchanged in 32% and decreased in 14%.

### Economic analysis results discussion

Estimating the economic impact of any intervention is complex and depends on the base assessments made and the context of the analysis which varies in each healthcare system. However, in the NHS, only cost directly attributable to the NHS itself can be included. The most recent and comprehensive analysis of the cost of breast cancer adjuvant chemotherapy in the NHS has been presented by Berdunov et al. [16] and has been used in our calculations. By convention, the cost of the Oncotype DX test itself must be its list price, whilst it is known that the NHS pays less for its block contract, but this figure is commercial in confidence. This means the figure shown is likely to be a significant underestimate of the savings.

What is more, this estimate does not consider the costs not directly attributable to the NHS such as the economic impact on the patient from the long-term psychological and physical effects of chemotherapy, the cost of travel for treatment, the costs of time off work required by the patient or their carers during treatment or the costs of dealing with the long-term complications of chemotherapy.

## Conclusions

This trial of 664 evaluable patients with 1–3 node positive early, HR+, HER2− breast cancer demonstrates that the use of Oncotype DX test in selecting patients for adjuvant chemotherapy results in a relative reduction in chemotherapy of 65%.

The trial also demonstrates an improvement of confidence in the final treatment selection by knowing the RS result for both oncologists and patients (oncologists: 55% improved, 33% unchanged and 12% reduced; patients: 71% improved, 22% unchanged and 7% reduced).

The economic analysis suggests a cost saving to the NHS of at least £787 per patient when Oncotype DX testing is available to the oncologists and at least £1100 in the subset of post-menopausal patients who were recruited after RxPONDER reported.

In summary, the use of Oncotype DX testing in early node-positive breast cancer reduces the suffering and inconvenience to the patients by sparing more than half of them chemotherapy which, in turn, then reduces the care demands on Oncology departments. It also reduces the costs of treatment so that NHS resources can be redistributed to other medical priorities.

### Supplementary information

Appendix

## Data Availability

The anonymised dataset is available from the corresponding author (simon_holt@mac.com).
