# Supplementary material for: A UK prospective multicentre decision impact, decision conflict and economic evaluation of the 21-gene assay in women with node+ve, hormone receptor+ve, HER2-ve breast cancer
Source: Br J Cancer. 2024 Feb 2;130(7):1149–56. doi: 10.1038/s41416-024-02588-9 (PMC10991515; doi:10.1038/s41416-024-02588-9)

Appendix to:

A UK prospective multicentre decision impact, decision conflict and economic evaluation of the 21- gene assay in women with node+ve, hormone receptor+ve, HER2-ve breast cancer

Figure 1: Recruitment by centre:

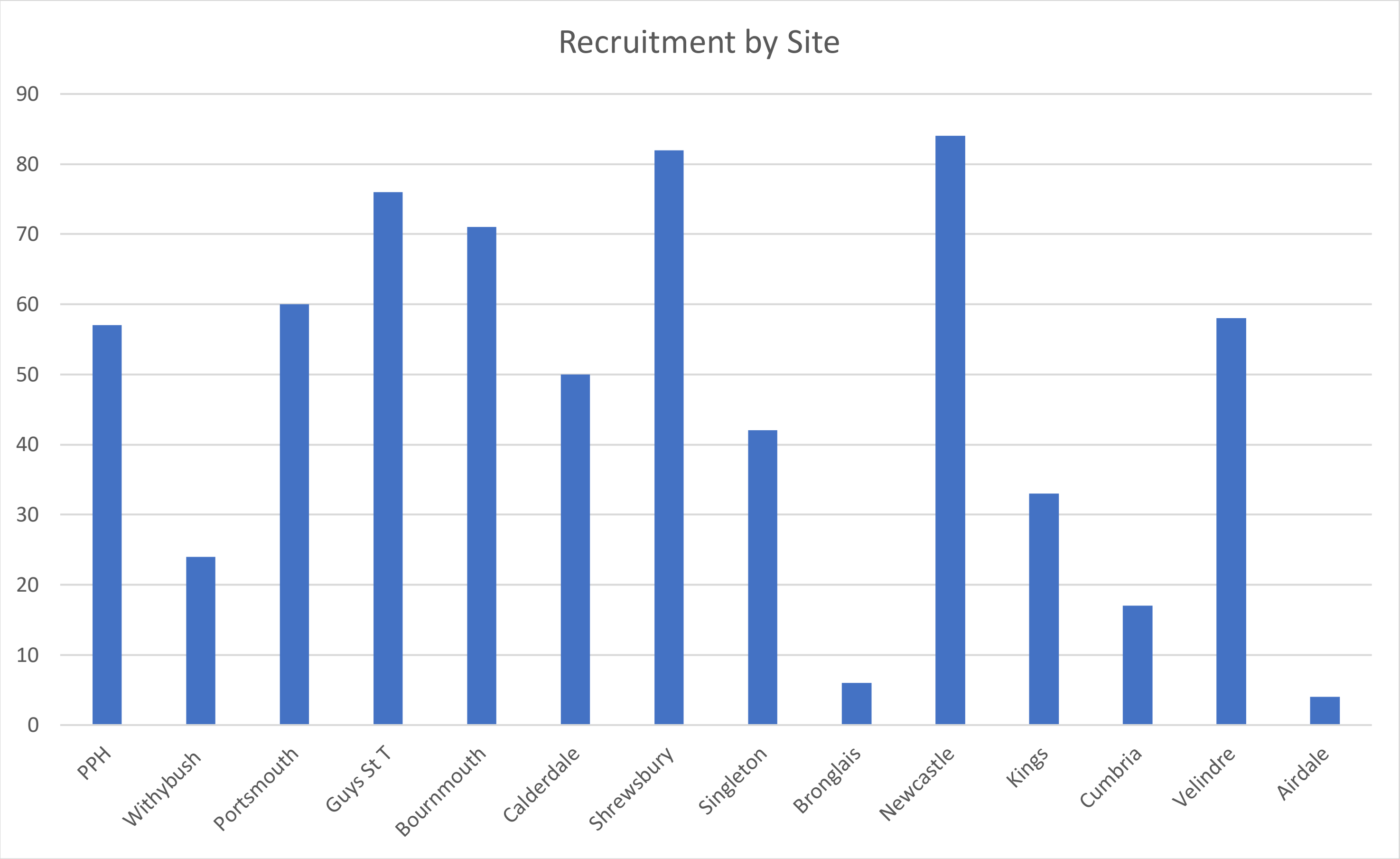

PPH – Prince Philip Hospital, Guys St T – Guy's and St Thomas'

Figure 2. Accrual rates by quarter.

Figure 2 summarises the accrual rate of patients by quarter. This demonstrates a fairly steady accrual rate with a small dip at the onset of the COVID-19 epidemic.

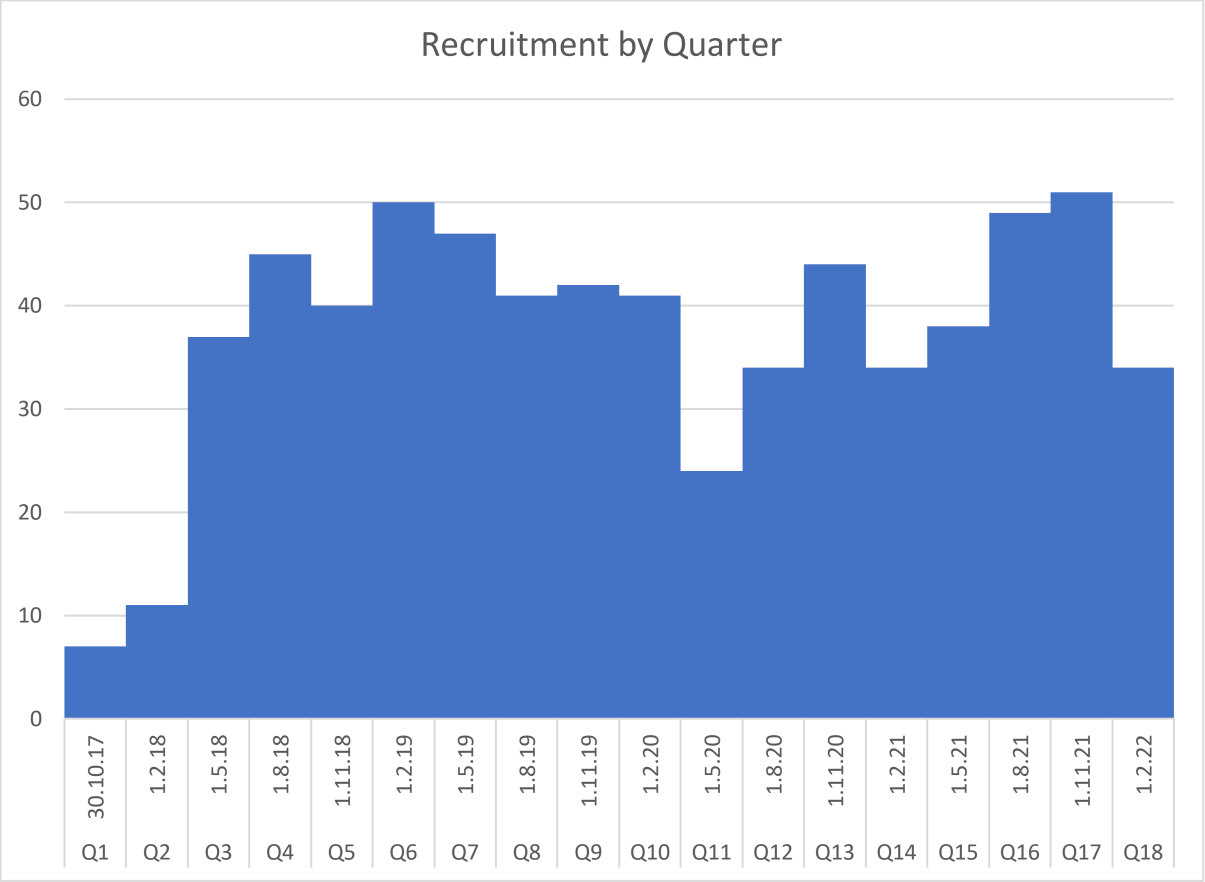

Figure 3. Age distribution of all patients and of the pre- and post-RxPONDER cohorts.

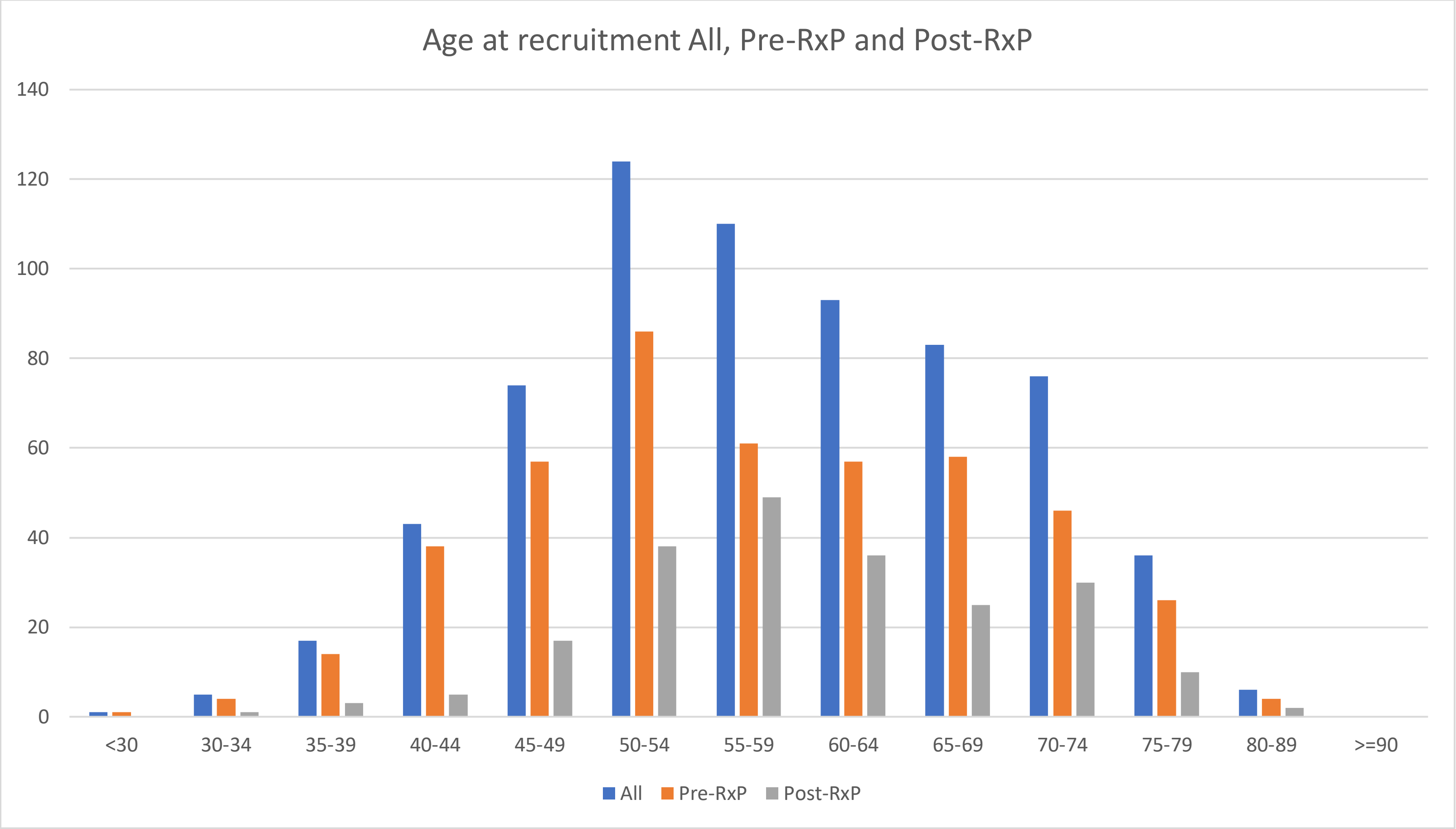

Table 1: Recurrence Score distributions (all, Pre- and Post-RxPONDER)

All RS	Overall (N, %)	Pre-RxPONDER (N, %)	Post-RxPONDER (N, %)
0 to 5	48 (7.2)	31 (6.9)	17 (7.9)
6 to 10	110 (16.6)	72 (16.0)	38 (17.8)
11 to 15	169 (25.5)	122 (27.1)	47 (22.0)
16 to 20	142 (21.4)	100 (22.2)	42 (19.6)
21 to 25	97 (14.6)	63 (14.0)	34 (15.9)
26 to 30	40 (6.0)	28 (6.2)	12 (5.6)
31 to 35	13 (2.0)	7 (1.6)	6 (2.8)
36 to 40	21 (3.2)	15 (3.3)	6 (2.8)
41 to 45	8 (1.2)	3 (0.7)	5 (2.3)
46 to 50	5 (0.8)	3 (0.7)	2 (0.9)
51 to 60	3 (0.5)	3 (0.7)	0 (0.0)
Over 61	8 (1.2)	3 (0.7)	5 (2.3)
Total	664	450	214

Figures 4, 5 and 6: Final Chemotherapy decision by Recurrence Score (All patients, Pre-RxPONDER and Post-RxPONDER)

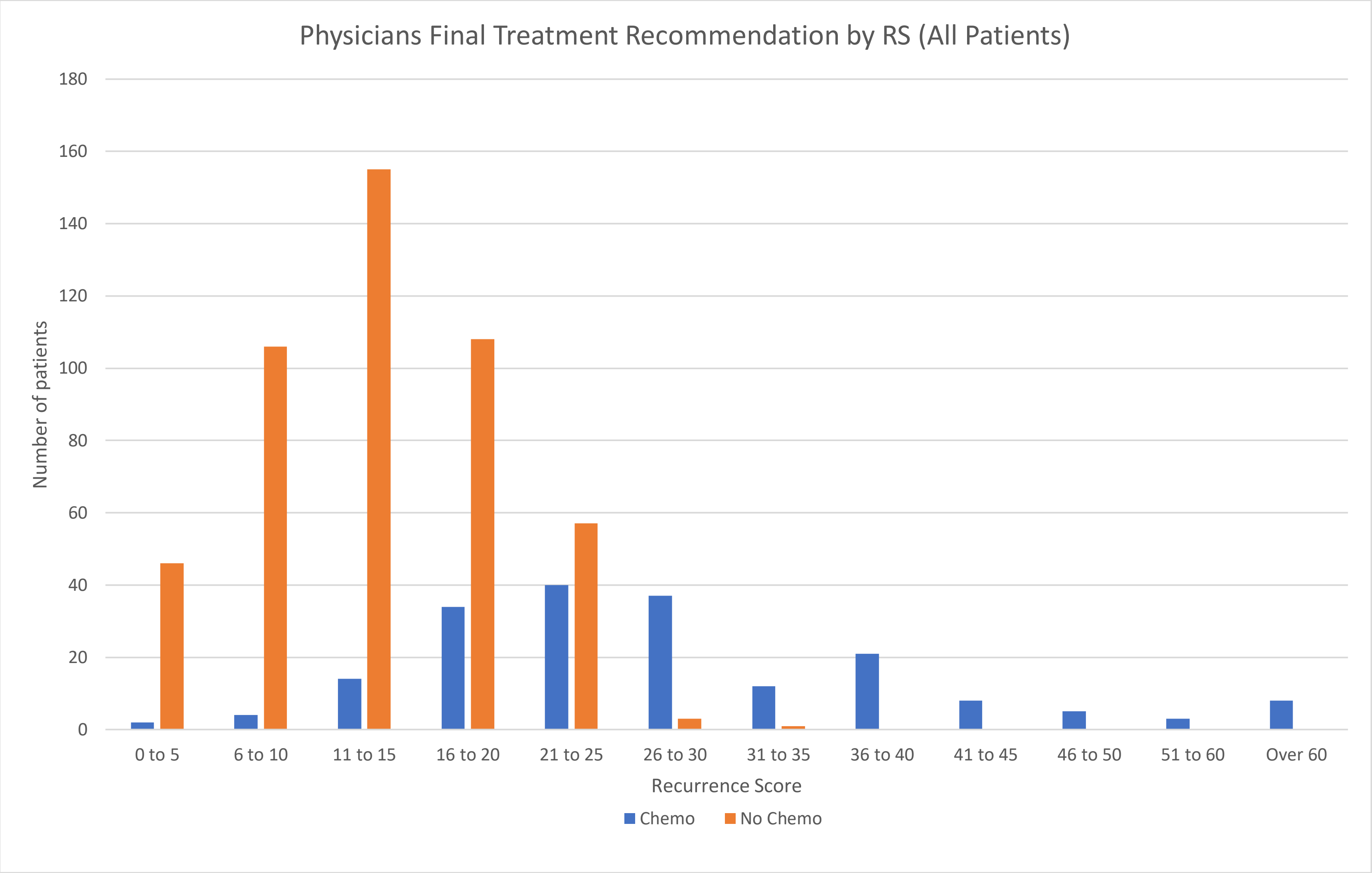

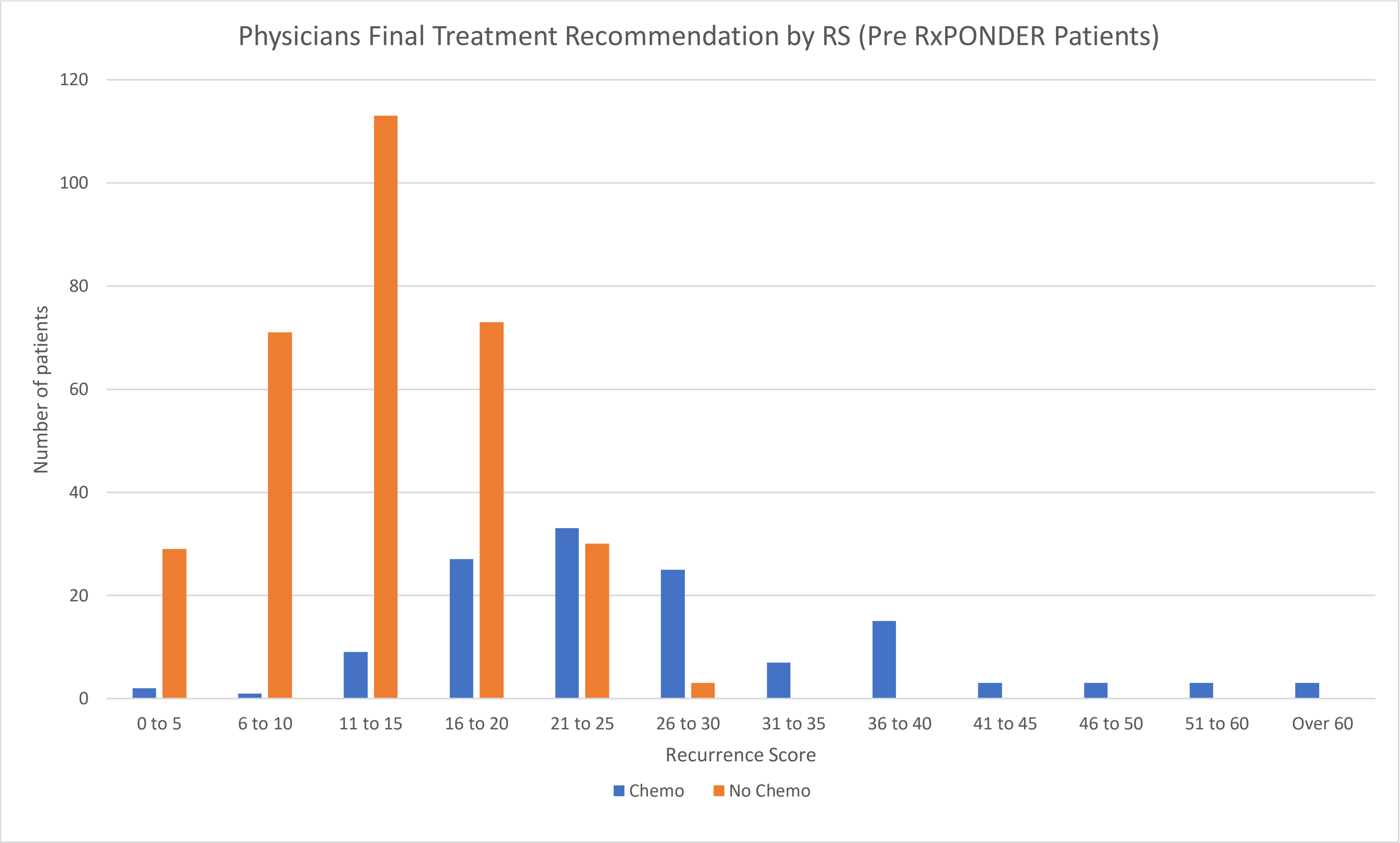
Physicians Final Treatment Recommendation by RS (Pre RxPONDER Patients)
Number of patients
0
20
40
60
80
100
120
0 to 5
6 to 10
11 to 15
16 to 20
21 to 25
26 to 30
31 to 35
36 to 40
41 to 45
46 to 50
51 to 60
Over 60
Recurrence Score
Chemo
No Chemo

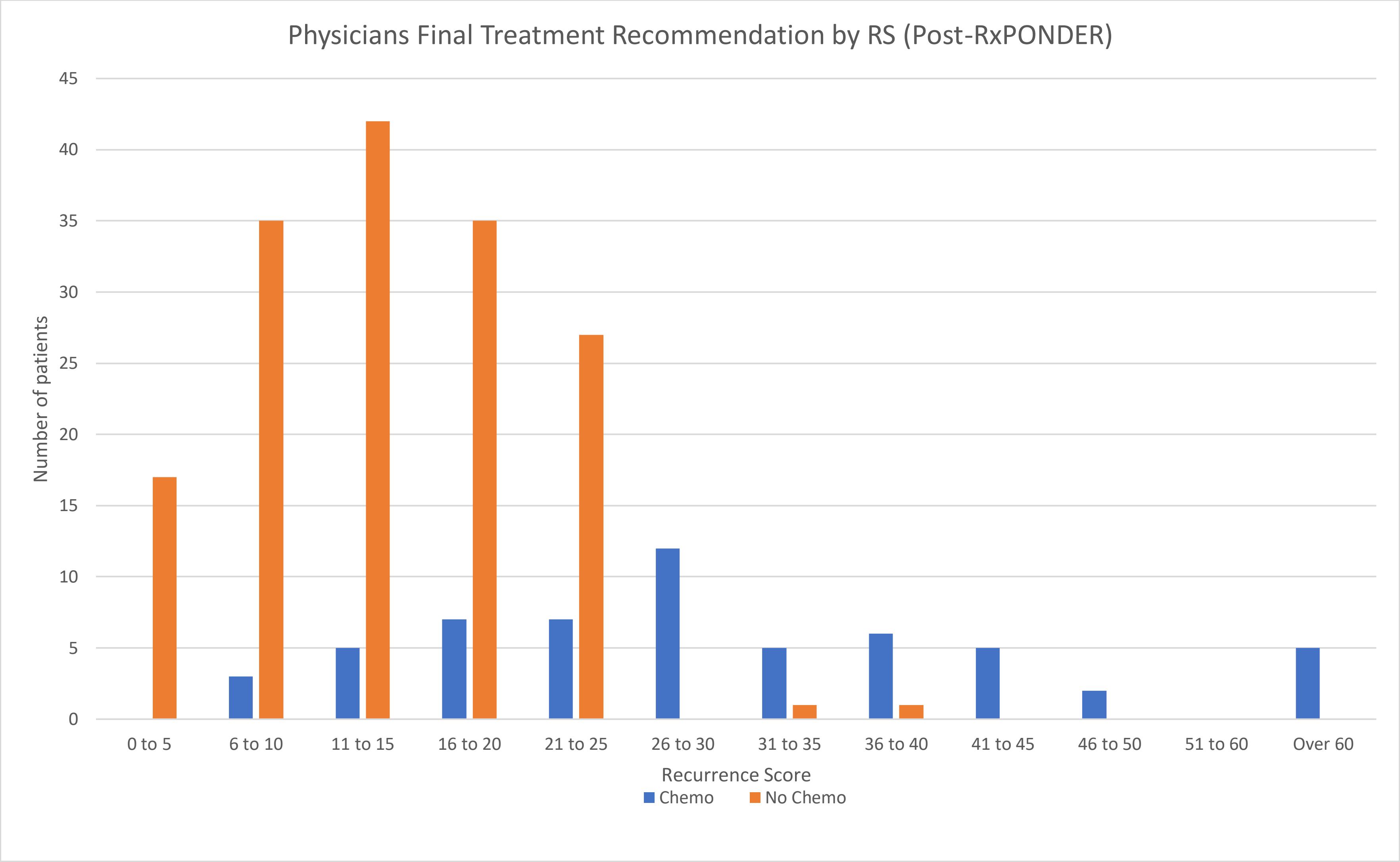

Physicians Final Treatment Recommendation by RS (Post-RxPONDER)
Number of patients
45
40
35
30
25
20
15
10
5
0
0 to 5
6 to 10
11 to 15
16 to 20
21 to 25
26 to 30
31 to 35
36 to 40
41 to 45
46 to 50
51 to 60
Over 60
Recurrence Score
Chemo
No Chemo

Table 2. Patients' Decision impact results. (All patients and for Pre- and Post-RxPONDER cohorts).

**PATIENT**			
Decision impact	Overall (N, %)	Pre-RxPONDER	Post-RxPONDER
CT Unchanged	87 (13.24)	70 (15.77)	17 (7.98)
HT Unchanged	52 (7.91)	36 (8.11)	16 (7.51)
CT+HT to HT	179 (27.25)	118 (26.58)	61 (28.64)
HT+HT to CT	7 (1.07)	6 (1.35)	1 (0.47)
UC to Chemo	78 (11.87)	45 (10.14)	33 (15.49)
UC to HT	253 (38.51)	169 (38.06)	84 (39.44)
UC unchanged	1 (0.15)	0 (0.0)	1 (0.47)
Total	657 (100)	444 (100)	213 (100)

CT = Chemotherapy, HT= Hormone therapy, UC= unchanged.
7 patients with missing post-assay treatment decision.

Figure 7. Change in Oncologists' treatment recommendations by RS result.

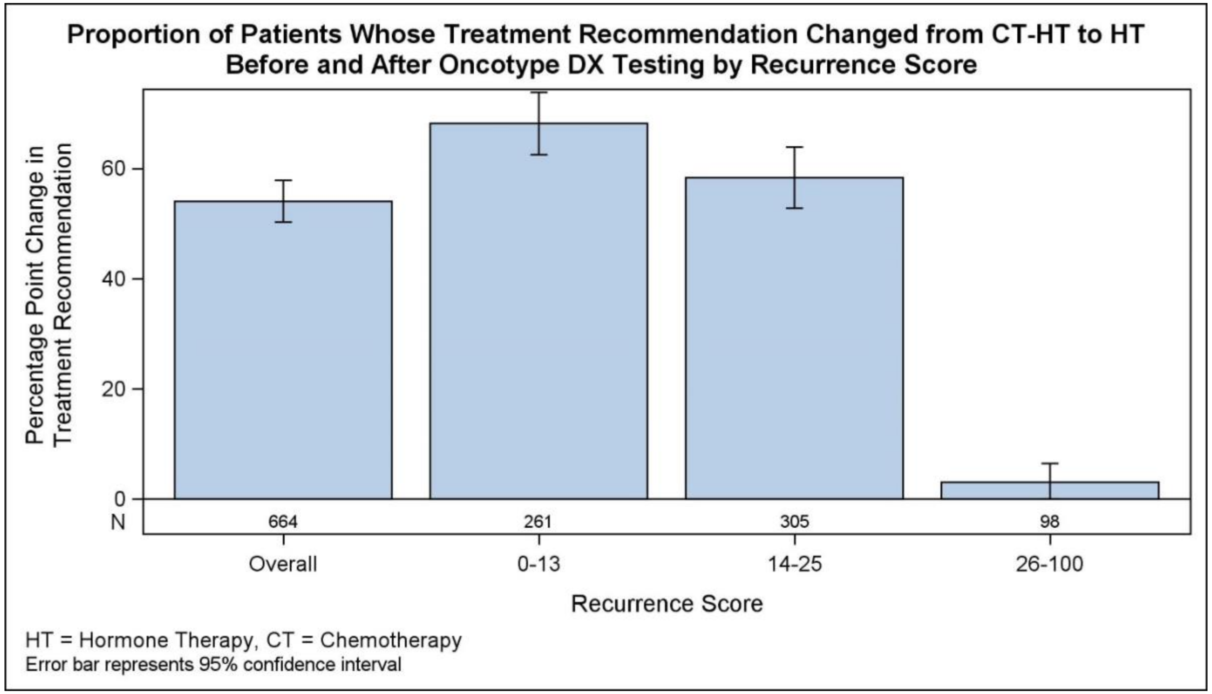

Figure 8. Change in Oncologists' treatment recommendations by number of nodes involved.

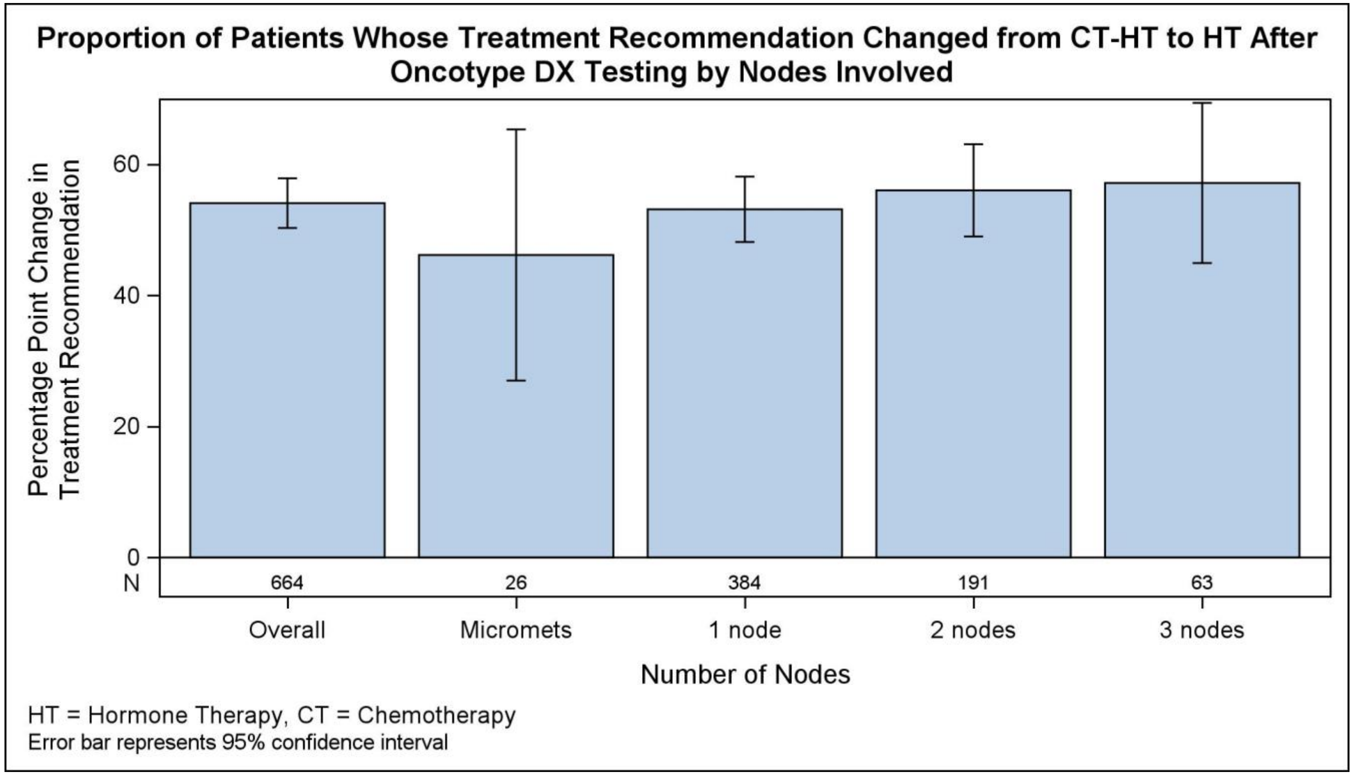

Supplement: Supplementary file 1 — Appendix [file 41416_2024_2588_MOESM1_ESM.pdf]
